# Epidemiological and clinical characteristics of respiratory viruses in 4403 pediatric patients from multiple hospitals in Guangdong, China

**DOI:** 10.1186/s12887-021-02759-0

**Published:** 2021-06-17

**Authors:** Yajie Zhang, Lin Qiao, Jinxiu Yao, Nan Yu, Xiaoping Mu, Shengqi Huang, Bo Hu, Weixuan Li, Feng Qiu, Fangyin Zeng, Cong Chen, Yuqiu Zhou, Bashan Zhang, Tian Cai, Weijia Wang, Xianjin Wu, Yiwen Zhou, Guochang Wang, Bo Situ, Shuling Lan, Na Li, Xiu Li, Zihua Li, Xin Li, Congrong Wang, Chao Yang, Pingfeng Feng, Hongxia Wang, Sijing Zhu, Yufeng Xiong, Min Luo, Wenjuan Shen, Xiumei Hu, Lei Zheng

**Affiliations:** 1 Department of Laboratory Medicine, Nanfang Hospital, Southern Medical University, Guangzhou, China; 2 Guangdong 999 Brain Hospital, Guangzhou, China; 3 Yangjiang People’s Hospital, Yangjiang, China; 4 Zhujiang Hospital, Southern Medical University, Guangzhou, China; 5 Guangdong Women and Children Hospital, Guangzhou, China; 6 Jiangmen Central Hospital, Jiangmen, China; 7 The Third Affiliated Hospital of Sun Yat-sen University, Guangzhou, China; 8 The First People’s Hospital of Foshan, Foshan, China; 9 Nanhai Hospital, Southern Medical University, Foshan, China; 10 The Fifth Affiliated Hospital of Southern Medical University, Guangzhou, China; 11 Central People’s Hospital of Zhanjiang, Zhanjiang, China; 12 Zhuhai Maternal and Child Health Hospital, Zhuhai, China; 13 Dongguan People’s Hospital, Dongguan, China; 14 Nanhai District People’s Hospital of Foshan, Foshan, China; 15 Zhongshan People’s Hospital, Zhongshan, China; 16 Central People’s Hospital of Huizhou, Huizhou, China; 17 Shenzhen Hospital of Southern Medical University, Shenzhen, China; 18 School of Economics, Jinan University, Guangdong Guangzhou, China; 19 Nanfang College of Sun Yat-Sen University, Guangdong Guangzhou, China; 20 The Seventh Affiliated Hospital, Sun Yat-Sen University, Guangdong Guangdong, China

**Keywords:** Acute respiratory infections, Prevalence, Seasonal, Respiratory viral infections, Multi-center, China

## Abstract

**Background:**

Acute respiratory infections (ARI) cause considerable morbidity and mortality worldwide, especially in children. Unfortunately, there are limited multi-center data on common viral respiratory infections in south China.

**Methods:**

A total of 4403 nasal swabs were collected from children in 10 cities in Guangdong, China in 2019. Seven respiratory viruses, influenza A virus (IFA), influenza B virus (IFB), respiratory syncytial virus (RSV), adenoviruses (ADV) and parainfluenza virus types 1–3 (PIV1, PIV2 and PIV3), were detected by direct immunofluorescence antibody assay. The personal information and clinical characteristics were recorded and analyzed.

**Results:**

The results showed that at least one virus was detected in 1099 (24.96 %) samples. The detection rates of RSV, IFA, ADV, PIV3, PIV1 and PIV2 were 7.13 % (314/4403), 5.31 % (234/4403), 4.02 % (177/4403), 3.04 % (134/4403), 1.70 % (75/4403) and 1.16 % (51/4403), respectively. The detection rate of RSV was highest in 0–6-month-old children at 18.18 % (106/583), while the detection rate of IFA was highest in 12–18-year-old children at 20.48 % (17/83). The total detection rates in winter and spring were 35.67 % (219/614) and 34.56 % (403/1166), higher than those in summer, 17.41 % (284/1631), and autumn, 19.46 % (193/992).

**Conclusions:**

RSV and IFA were the main respiratory viruses in children. With increasing age the detection rate of RSV decreased in children, but the trends for the detection rates of IFA and IFB were the opposite. This study provided the viral etiology and epidemiology of pediatric patients with ARI in Guangdong, China.

**Supplementary Information:**

The online version contains supplementary material available at 10.1186/s12887-021-02759-0.

## Background

Acute respiratory infections (ARI) including the common cold, otitis media, acute bronchiolitis, pharyngitis, and pneumonia are one of the main causes of morbidity and mortality worldwide, especially in children [1]. Common pathogens causing ARI include viruses, bacteria and fungi. Respiratory syncytial virus (RSV), parainfluenza viruses (PIV), influenza A virus (IFA), influenza B virus (IFB) and adenoviruses (ADV) are the main respiratory viruses found in children [2]. Stringent diagnostic criteria are used to confirm the diagnosis of ARI, but empiric treatment for ARI is still common. Given that the symptoms of respiratory infections are usually clinically similar, it is difficult to distinguish viral infections from bacterial infections based on symptoms [3]. Therefore, the accurate and timely detection of respiratory tract pathogens in the laboratory is of great significance for correct diagnosis.

The prevalence of each respiratory pathogen varies from region to region, possibly owing to differences in climate, culture, and geography. A better understanding of the epidemiological and clinical characteristics of respiratory infections leading to ARI is critical for the successful implementation of prevention, control, and treatment strategies. Although some studies on the epidemiology of respiratory infections including ARI have recently been reported for local areas of China [4, 5], there have been few articles reporting the epidemiological and clinical characteristics of respiratory viruses from multiple hospitals. To better understand the nature of respiratory infections in children, we analyzed epidemiological and clinical characteristics of respiratory infections in pediatric patients aged 0–18 years from 16 hospitals in Guangdong, China in 2019.

## Methods

### Study population and design

We conducted a retrospective, observational study that included emergency, outpatient and inpatient pediatric patients from 16 hospitals in Guangdong, China. All pediatric patients with ARI admitted to from January 1 to December 31, 2019 were screened for eligibility. Inclusion criteria were age < 18 years, admission to any of the hospitals for < 24 h, and agreement to provide written informed consent. The participating hospitals were located in 10 cities of Guangdong province (Additional file 1). Pediatric patients with symptoms and signs of respiratory tract infection, such as fever, cough, expectoration, sore throat, nasal congestion, nasal discharge, shortness of breath, abnormal breath sounds on auscultation, chest pain and lower respiratory signs, and inflammatory injury in the lungs observed upon imaging analysis, were defined as having ARI.

The following data were obtained from enrolled participants: age, gender, time of year, clinical symptoms and laboratory information. The clinical characteristics and laboratory information were collected and analyzed.

### Sample collection and laboratory processing

All admitted pediatric patients with symptoms of respiratory tract infection attending 16 hospitals had nasal swabs or a bronchial aspirate taken for the study of respiratory viruses. Respiratory viruses were identified by direct immunofluorescence antibody assay using the D3 Ultra DFA virus identification reagent (Diagnostic Hybrids, Inc., USA), including IFA, IFB, ADV, RSV, PIV1, PIV2, and PIV3. The assay was conducted according to the manufacturer’s instructions. All the medical staff and laboratory staff of the participating hospitals had received standardized training. Their patients provided clinical information for research purposes by completing a standard questionnaire under the guidance of the trained clinician. All methods were performed in accordance with the relevant guidelines and regulations.

### Statistical methods

Statistical analyses were performed using IBM SPSS statistical software version 20 for Windows (IBM Corp., Armonk, New York, USA). Descriptive frequencies were presented as mean ± standard deviation and proportions. Chi-square tests and Fisher’s exact tests were used for comparisons between groups in terms of categorical variables wherever appropriate. Multivariate logistic regression analysis was performed to identify factors associated with the outcomes of interest. A *P*-value < 0.05 was considered statistically significant.

## Results

### Demographic Characteristics and Overall Prevalence of Respiratory Viruses

**Fig. 1:**
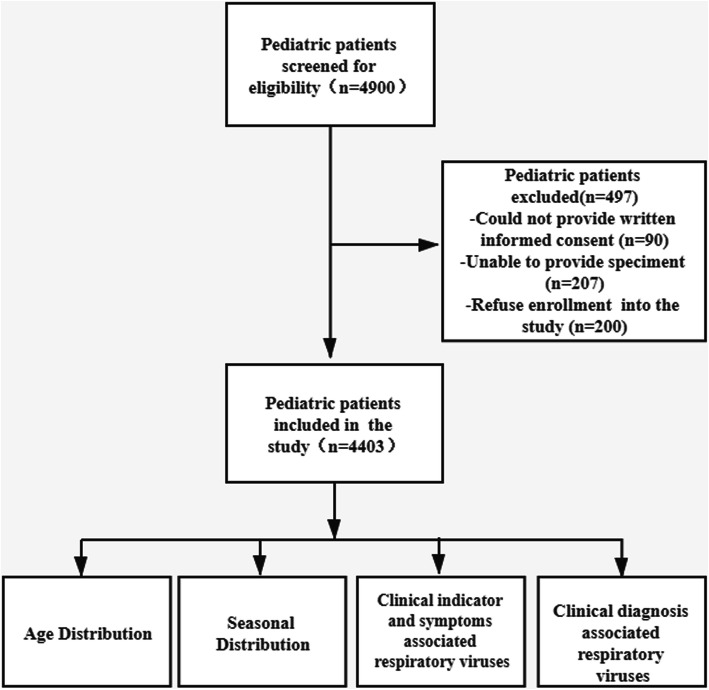
Study design

A total of 4403 nasal swab specimens from pediatric patients with ARI in Guangdong, China in 2019 were analyzed (Fig. 1). Overall, 2520 (57.23 %) of the pediatric patients were male and 1883 (42.77 %) were female (*P* < 0.05). The mean age was 4 years (range 0 months to 18 years), and the median age was 6 years.

**Fig. 2:**
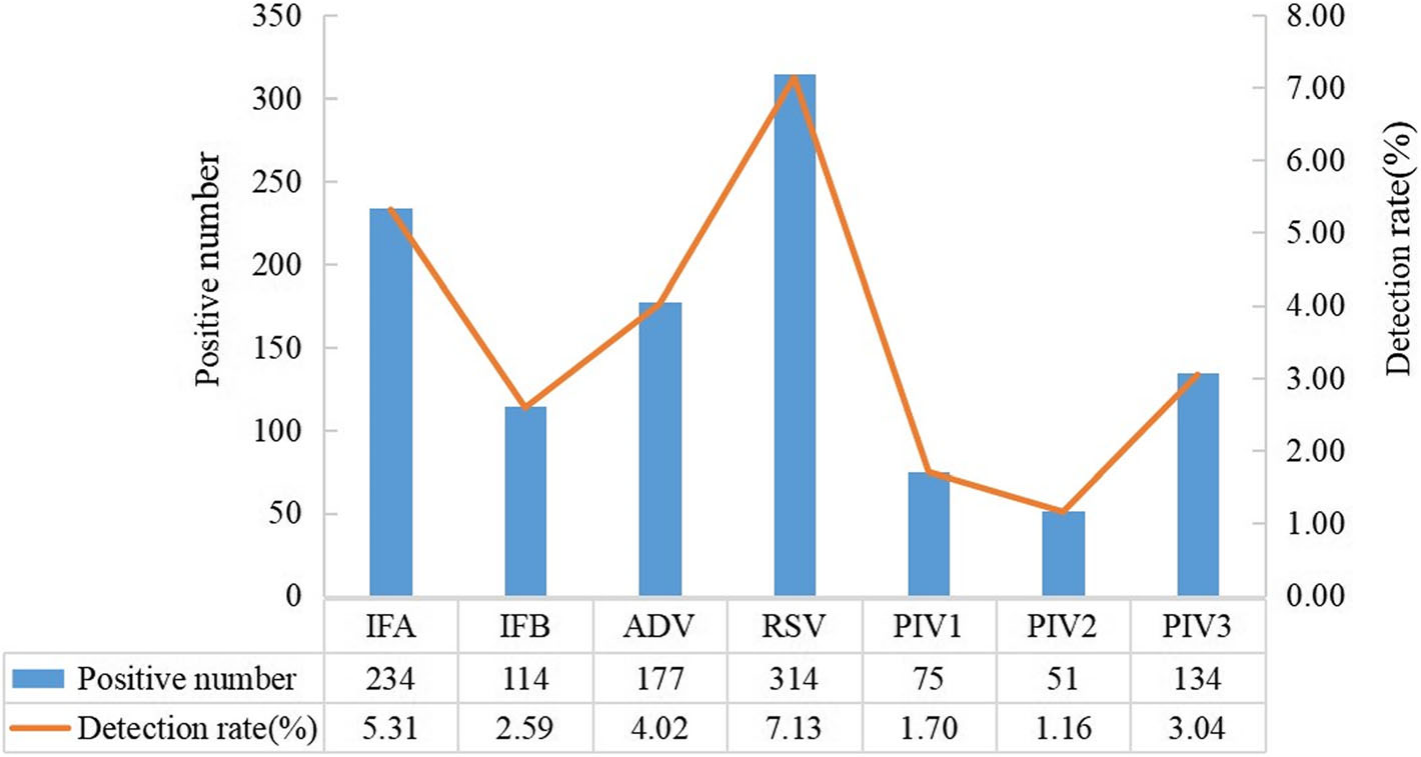
The detection rate of respiratory viruses in pediatric patients with ARI in Guangdong, China, 2019

The total virus detection rate of all cases was 24.96 % (1099/4403); 1076 (24.44 %) of the specimens showed single infections and 23 (0.52 %) of the specimens showed multiple infections. Among the respiratory viruses, the detection rate of RSV was 7.13 % (314/4403), which was the highest, followed by IFA 5.31 % (234/4403), ADV 4.02 % (177/4403), PIV3 3.04 % (134/4403), PIV1 1.70 % (75/4403) and PIV2 1.16 % (51/4403) (Fig. 2). The total virus detection rate in males was 25.40 % (640/2520), and it was 24.38 % (459/1883) in females (*P* > 0.05).

### Age-related Prevalence of Respiratory Viruses

**Fig. 3:**
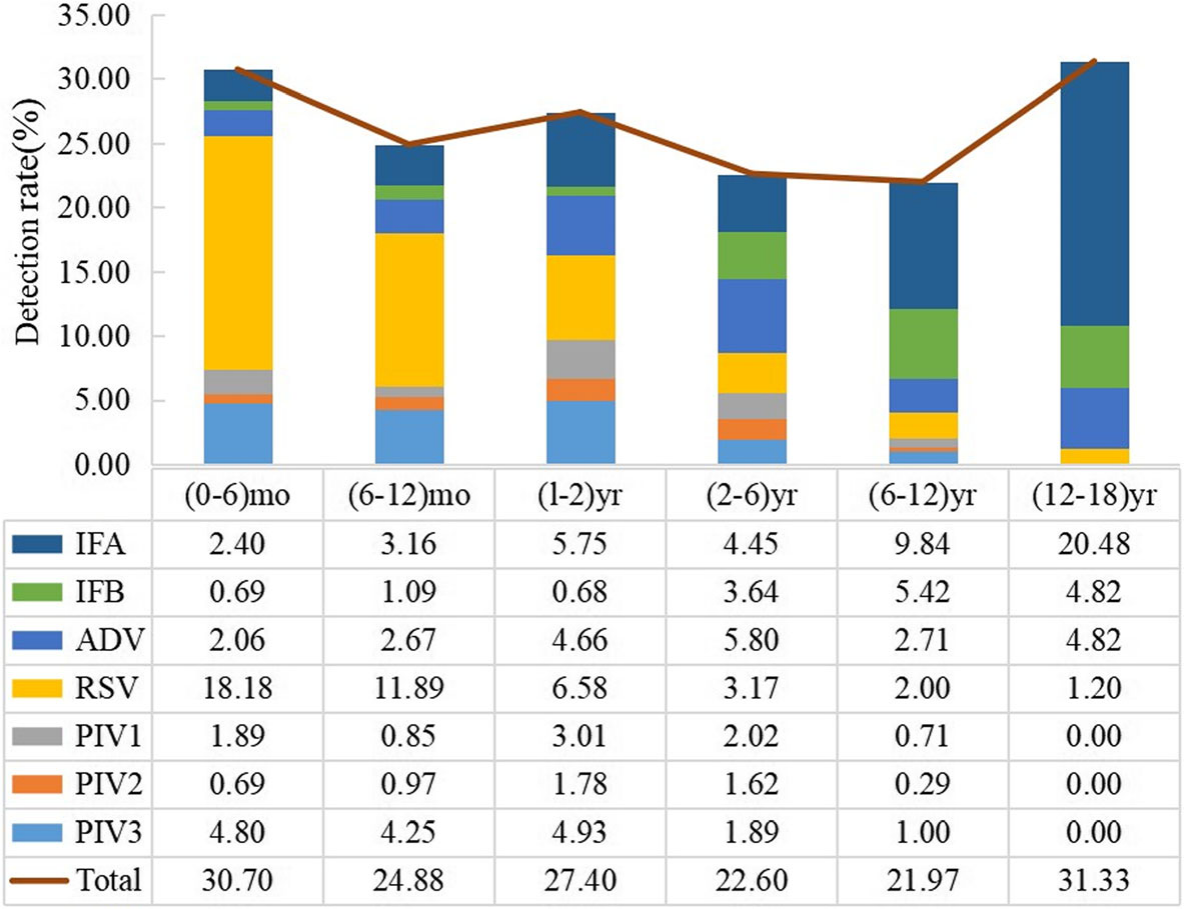
Age distribution of respiratory viruses in pediatric patients with ARI in Guangdong, China, 2019. Note: There was statistically significant associations between respiratory virus (IFA, IFB, ADV, RSV, PIV1, PIV2 and PIV3) and age group (*P* < 0.05, for all comparisons).

The distribution of respiratory viruses among different age groups is shown in Fig. 3. The virus detection rates in the 0–6-month-old group, 6–12-year-old group, 1–2-year-old group, 2–6-year-old group, 6–12-year-old group and 12–18-year-old group were 30.70 % (179/583), 24.88 % (205/824), 27.40 % (200/730), 22.60 % (335/1482), 21.97 % (154/701) and 31.33 % (26/83), respectively. In the 0–6-month-old group, the top two detected viruses were RSV (18.18 %, 106/583) and PIV3 (4.80 %, 28/583), and in the 6–12-month-old group, the two most common viruses were also RSV (11.89 %, 98/824) and PIV3 (4.25 %, 35/824). In the 1–2-year-old group, the top two respiratory viruses were RSV (6.58 %, 48/730) and IFA (5.75 %, 42/730). In the 2–6-year-old group, the pathogen with the highest detection rate was ADV (5.80 %, 86/1482), followed by IFA (4.45 %, 66/1482). In the 6–12-year-old group, IFA (9.84 %, 69/701) and IFB (5.42 %, 38/701) were the top two detected viruses. Among the 12–18-year-old group, IFA (20.48 %, 17/83) was the most common respiratory virus, followed by ADV (4.82 %, 4/83) and IFB (4.82 %, 4/83).

Two distinct trends in virus detection rates were observed in different age groups. The detection rate of RSV decreased with age and detection of influenza A and B increased with age. PIVs were more frequently detected in children younger than 6 years. Among them, PIV3 was predominantly obtained from patients under 2 years old. PIV1, PIV2 and ADV showed a peak of detection in the 1–6-year-old group. The detection rate of respiratory viruses differed significantly among the age groups (*P <* 0.05).

### Seasonal Distribution

**Fig. 4:**
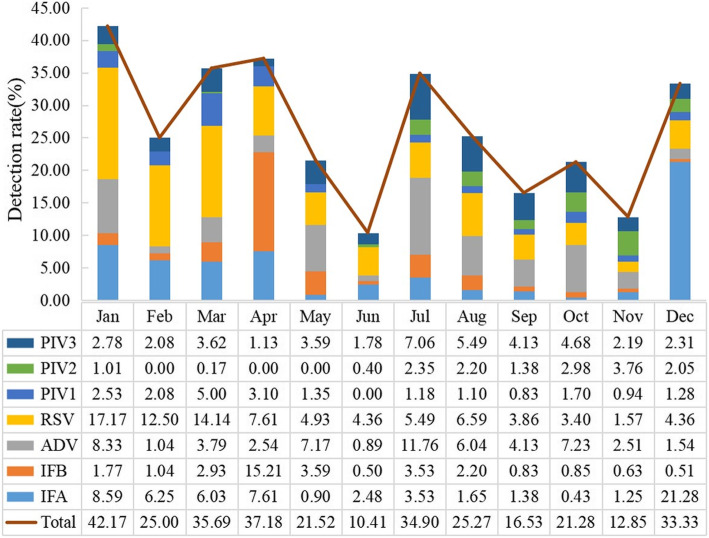
Seasonal distribution of respiratory viruses in pediatric patients with ARI, 2019. Note: There were statistically significant associations between respiratory virus (IFA, IFB, ADV, RSV, PIV1, PIV2 and PIV3) and month (*P* < 0.05, for all comparisons).

In this study, we analyzed the virus detection rates from January 2019 to December 2019. The results were divided into four seasons by combining astronomical seasons with climatic seasons in Guangdong province, China: spring (March, April and May), summer (June, July and August), autumn (September, October and November) and winter (December, January and February). As shown in Fig. 4, the virus detection rates in winter and spring (from December to May) were 35.08 % (221/630) and 37.13 % (398/1072), which were significantly higher than those in summer, at 16.99 % (285/1677), and autumn, at 19.04 % (195/1024) (*P* < 0.05). The virus detection rate was highest in January 42.17 % (167/396), followed by April 37.18 % (132/355), while the lowest virus detection rate was in June, at 10.41 % (105/1009).

For RSV, three peak months were identified, in January 17.17 % (68/396), March 14.14 % (82/580) and February 12.50 % (12/96). For ADV, three peak months were identified, in July 11.76 % (30/255), January 8.33 % (33/396) and October 7.23 % (17/235). The detection rate of IFA peaked in December, at 21.28 % (83/390), followed by January, at 8.59 % (34/396). The detection rate of IFB was highest in April, at 15.21 % (54/355). In the seasonal distribution of viruses, the highest detection rates of PIV1, PIV2 and PIV3 were in March 5.00 % (29/580), November 3.76 % (12/319) and July 7.06 % (18/255), respectively. The detection rates of respiratory viruses differed significantly among different months (*P <* 0.05).

### Clinical Indicators and Symptoms Associated with Respiratory Viruses

In this study, we statistically analyzed pediatric patients who had clinical data in their medical record. The mean respiratory rate of PIV1 positive pediatric patients was 36.18 ± 13.00 breaths/min, significantly faster than that of PIV1 negative pediatric patients (*P <* 0.05). The mean value of maximum body temperature of IFA positive pediatric patients was 39.46 ± 0.55℃, which was considerably higher than that of IFA negative pediatric patients. The shortest and longest duration of fever were shown by PIV1 positive pediatric patients (3.46 ± 2.81 days) and PIV2 positive pediatric patients (6.00 ± 5.35 days) (Table 1 and Additional file 2).

**Table 1:** Clinical Indicators and Clinical Symptoms Associated with Respiratory Viruses Positive

Characteristics	IFA	IFB	ADV	RSV	PIV1	PIV2	PIV3
Clinical indicators							
Respiratory rate (breaths/min)	28.33 ± 5.77	22.00 ± 11.04	30.50 ± 12.02	30.17 ± 9.81	36.18 ± 13.00	26.33 ± 6.50	24.33 ± 8.50
Duration of fever (days)	5.00 ± 6.27	4.23 ± 3.72	4.60 ± 4.63	4.03 ± 2.83	3.46 ± 2.81	6.00 ± 5.35	5.42 ± 4.35
Check body temperature (℃)	37.88 ± 0.94	33.07 ± 12.43	32.50 ± 14.52	30.71 ± 14.93	35.56 ± 8.67	30.04 ± 16.81	23.90 ± 18.96
Maximum body temperature (℃)	39.46 ± 0.55	36.54 ± 10.53	35.88 ± 11.74	33.51 ± 13.90	39.04 ± 0.59	29.77 ± 19.85	27.20 ± 18.88
Clinical Symptoms^a^							
Cyanosis	0(0.00)	0(0.00)	1(0.82)	5(1.95)	1(2.00)	0(0.00)	0(0.00)
Fever	112(94.10)	34(85.00)	110(89.40)	167(70.50)	23(79.30)	17(89.50)	60(61.20)
Shivering	8(6.67)	1(2.04)	9(7.38)	1(0.40)	1(2.00)	0(0.00)	0(0.00)
Nasal congestion	21(17.50)	3(6.12)	20(16.39)	47(18.36)	4(8.00)	2(13.30)	19(20.21)
Nasal discharge	18(15.00)	3(6.12)	22(18.03)	46(17.97)	8(15.70)	4(26.700)	17(18.48)
Sore throat	6(5.00)	2(4.08)	4(3.28)	2(0.78)	0(0.00)	0(0.00)	0(0.00)
Cough	80(66.67)	37(75.51)	89(72.95)	224(87.50)	43(86.00)	18(100.00)	88(87.81)
Chest tightness	1(0.83)	0(0.00)	1(0.82)	8(3.13)	1(2.00)	1(5.00)	2(2.17)
Shortness of breath	6(5.00)	2(4.08)	13(10.66)	23(8.98)	1(2.00)	0(0.00)	8(870)
Nausea	1(0.97)	0(0.00)	0(0.00)	0(0.00)	1(0.2.00)	0(0.00)	0(0.00)
Abdominal pain	6 (6.19)	1(2.04)	0(0.00)	1(0.39)	0(0.00)	0(0.00)	0(0.00)
Neurological symptoms	6 (6.19)	1(2.04)	40.34)	2(0.78)	1(2.00)	0(0.00)	1(1.09)

Note: ^a^: case (%)

In this study, fever was the most common clinical symptom and was identified in 75.88 % (2136/2815) of pediatric patients with ARI, followed by cough 73.29 % (2063/2815), nasal discharge 13.39 % (377/2815) and nasal congestion 11.90 % (335/2815).

Symptoms associated with infection of IFA were fever, shivering, nasal congestion, abdominal pain and neurological symptoms (*P <* 0.05). Compared with the 12–14-year age group, younger patients (< 12 years) had a lower risk of being IFB positive. Infection of ADV was associated with fever, shivering and shortness of breath (*P <* 0.05). Only cough was associated with RSV infection. Laboratory confirmation of PIV1 and PIV3 was associated with absence of fever. No clinical symptoms were associated with PIV2 infection (*P* > 0.05) (Tables 1 and 2 and Additional file 2).

**Table 2:** Multivariate Logistic Regression Analysis of Clinical characteristics associated with respiratory viruses

	IFA		IFB		ADV		RSV		PIV1		PIV2	PIV3	
	OR(95 %CI)	*P*	OR(95 %CI)	*P*	OR(95 %CI)	*P*	OR(95 %CI)	*P*	OR(95 %CI)	*P*	*P*	OR(95 %CI)	*P*
Sex		NS		NS		NS		NS		NS	NS		NS
Age groups													
(0–6)mo		NS	0.049 (0.010–0.235)	0.000		NS		NS		NS	NS		NS
(6–12)mo		NS	0.043 (0.010–0.186)	0.000		NS		NS		NS	NS		NS
(1–2)yr		NS	0.016 (0.002–0.149)	0.000		NS		NS		NS	NS		NS
(2–6)yr		NS	0.176 (0.056–0.552)	0.003		NS		NS		NS	NS		NS
(6–12)yr		NS	0.205 (0.060–0.697)	0.011		NS		NS		NS	NS		NS
(12–18)yr		NS	-^a^			NS		NS		NS	NS		NS
Clinical Symptoms													
Cyanosis		NS		NS		NS		NS		NS	NS		NS
Fever	3.847 (1.836–8.061)	0.000		NS	1.635 (1.006–2.659)	0.047		NS	0.305 (0.167–0.556)	0.000	NS	0.550 (0.351–0.863)	0.009
Shivering	2.733 (1.173–6.366)	0.020		NS	3.251 (1.514–6.981)	0.002		NS		NS	NS		NS
Nasal congestion	2.314 (1.200-4.461)	0.012		NS		NS		NS		NS	NS		NS
Nasal discharge		NS		NS		NS		NS		NS	NS		NS
Sore throat		NS		NS		NS		NS		NS	NS		NS
Cough		NS		NS		NS	2.152 (1.399–3.308)	0.000		NS	NS		NS
Chest tightness		NS		NS		NS		NS		NS	NS		NS
Shortness of breath		NS		NS	2.838 (1.497–5.38)	0.001		NS		NS	NS		NS
Nausea		NS		NS		NS		NS		NS	NS		NS
Abdominal pain	2.809 (1.126–7.011)	0.027		NS		NS		NS		NS	NS		NS
Neurological symptoms	3.642 (1.450–9.145)	0.006		NS		NS		NS		NS	NS		NS

Note: For each virus, an independent multivariate regression analysis was performed (i.e. whatever the virological result, positive or negative)

^a^: Reference group; *NS* no significant.

### Clinical Diagnosis Associated with Respiratory Viruses

The clinical diagnosis was divided into upper respiratory tract infection (URTI), lower respiratory tract infection (LRTI) and severe pneumonia. The virus detection rates in patients with URTI, LRTI, and severe pneumonia were 31.53 % (257/815), 23.20 % (301/1297), and 35.94 % (23/64), respectively.

**Fig. 5:**
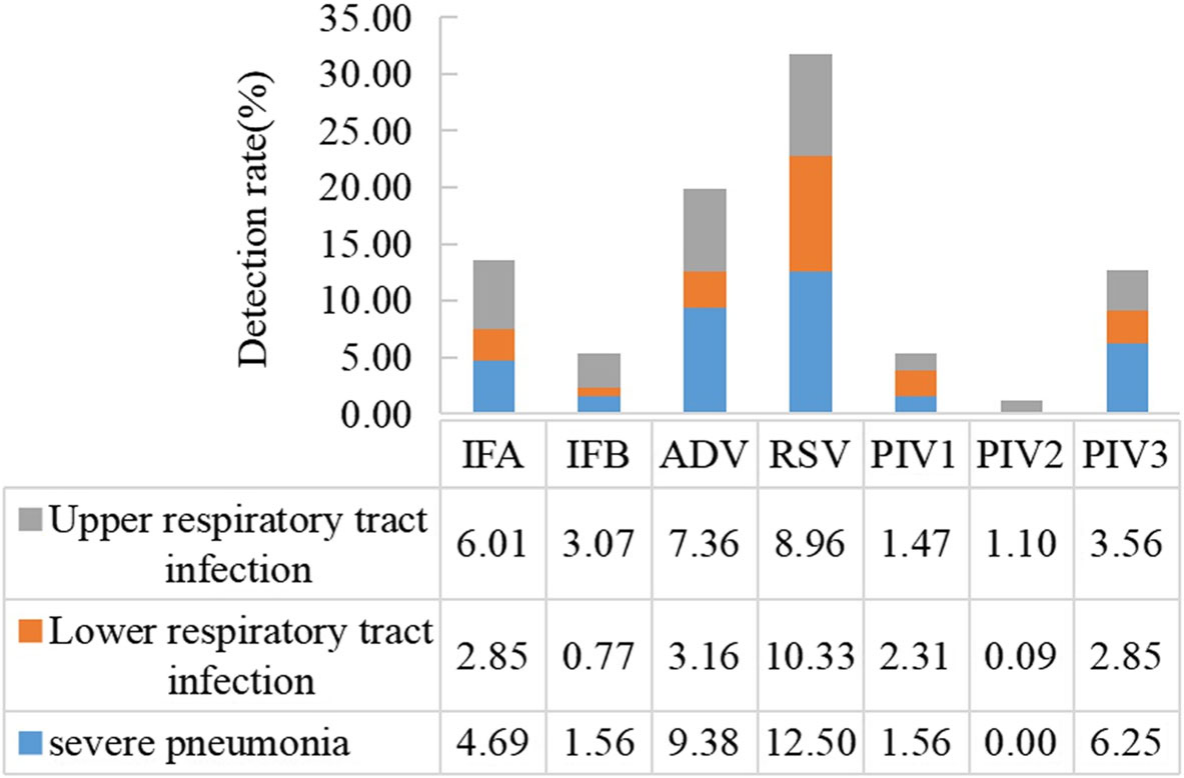
Clinical diagnosis of respiratory viruses in pediatric patients with ARI in Guangdong, China, 2019

The detection rates of IFA and IFB in URTI were 6.01 % (49/815) and 3.07 % (25/815), higher than those in LRTI (*P <* 0.05). The pediatric patients who were infected with IFA and IFB were more likely to be diagnosed with URTI (Fig. 5). Pediatric patients infected with ADV were more likely to be diagnosed with severe pneumonia 9.38 % (6/64) (*P <* 0.05). RSV mainly caused severe pneumonia and LRTI, with detection rates of 12.50 % (8/64) and 10.33 % (134/1297), respectively. PIV3 caused a higher proportion of severe pneumonia (6.25 %, 4/64).

## Discussion

Acute respiratory infection (ARI) is a common clinical disease and defining the cause is deceptively difficult [6]. Accurate and rapid identification of pathogens causing acute respiratory infection is essential for the application of appropriate antiviral therapy and the prevention of overuse of antibiotics. Although there are many reports of retrospective analyses of respiratory virus infection in Guangdong, China, few articles report comprehensive analyses from multiple hospitals. In this study, 4403 pediatric patients aged 0–18 years were recruited from 16 hospitals in 10 cities of Guangdong, China in 2019, namely Guangzhou, Zhuhai, Shenzhen, Dongguan, Huizhou, Zhanjiang, Zhongshan, Yangjiang, Jiangmen and Foshan (Additional file 1).

In our study, the detection rate of respiratory viruses in children with ARI was 24.96 %. In other studies, the rates ranged from 24.5 to 72.3 % in other regions of China and other countries [4, 5, 7–9], which indicates the complexity and diversity of ARI etiology. RSV and IFA were the two most frequently detected viruses in this study. This result is in good agreement with some previous studies [9–11], but differs from other studies [12, 13]. Our study indicated that RSV was the dominate cause of respiratory infection in children and the detection rate of RSV decreased with age, owing to the maturation of the immune system [14]; this finding was similar to previous reports [9, 11, 15]. IFA was the second most common respiratory virus. Influenza virus prevalence increased with age, which agreed with previous reports [8, 11], but differed from another study [9], and may be due to a greater opportunity for transmission within day care or preschool and more frequent social mixing by older children and those of school age [16]. In this study, the detection rate of ADV was the third highest, and it was more likely to infect pre-adolescents (2–12 years old) (*P* < 0.05); it is considered to be an important cause of respiratory tract infections in children [17]. Owing to the variability in virus transmission dynamics among humans, such as the reproductive number (R0), the rate of mutation accumulation, the duration of immunity, and the presence of cross-protection [8], the age distribution of these viruses shows diversity which may contribute to the determination of appropriate pediatric care and disease diagnosis.

ARI is frequently seasonal, particularly in regions with temperate climates [18]. In the Northern Hemisphere, respiratory viruses are reported to be more active from November to March [19]. In our study, the same trend was shown: the virus detection rate in winter (December, January, February) and spring (March, April, May) was higher than that in summer and autumn. However, in February, the detection rate of the virus was significantly lower than that of the other 2 months (December and January). A possible reason might be that the Chinese Lunar New Year occurs in February, and many people go home for the Spring Festival holiday. In addition, Guangdong Province is a large province with a floating population, and in February the population aggregation in public places decreases significantly. Infection with IFA, IFB and RSV was seasonal, with high incidence in winter and spring, consistent with findings reported in Spain, the USA [20, 21], and Shanghai, China [4], but different from Qingdao, China [18], and other counties [9, 22, 23], which suggested that there were regional differences in virus prevalence. The detection rate of ADV peaked in July 2019 (10.51 %), which was similar to what has been found in Beijing, China [24], where ADV was primarily detected in the summer; however, in Kuwait, peaks occurred in November and March [25]. In our study, many cases of PIV2 infection were detected in the autumn-winter season, while PIV3 showed clear seasonality with yearly outbreaks in the spring-summer season, consistent with a previous report [26], but PIV1 infections peaked in spring, not in autumn. Seasonal variation may correlate with viral interference [27]; China’s complex climate, including low temperature, humidity, and precipitation, and geography, as well as its diverse socio-economic and demographic characteristics, have led to different patterns of seasonal virus infections, suggesting that more studies are needed to guide vaccination times in the south of China [28].

In our study, it was shown that the most common clinical symptoms were fever and cough in children with acute respiratory infection, consistent with a previous report [29]. Our data suggested that cough was associated with RSV detection. RSV infection was more likely to be associated with lower respiratory tract infection (10.33 %) and severe pneumonia (12.5 %), as previously reported [13, 30], which indicated that it was necessary to pay more attention to RSV infection. Symptoms associated with infection of IFA were fever, shivering, nasal congestion, abdominal pain and neurological symptoms. More influenza cases had high fever at hospital admission than those without influenza [31]. This suggested that, in the influenza epidemic season, children with high fever should be first assessed for IFA infection, and need active treatment to reduce the possibility of febrile seizures. PIV1 and PIV3 were associated with absence of fever, which indicated that if there was no fever a patient may be infected with PIV1 or PIV3. However, the symptoms of respiratory virus infections are usually clinically similar, so it is very important that the detection of pathogen infection through direct or indirect fluorescence and other laboratory tests be used to provide clinicians with diagnostic evidence.

This study reflected the respiratory virus infections in pediatric patients with ARI in Guangdong, China during 2019. The large data set from 16 hospitals provided an adequate database, which allowed us to draw meaningful conclusions regarding the agents.

Regrettably, some other common respiratory viruses and bacteria were not included in our study and the pathogens detected by direct immunofluorescence antibody assay are extremely limited. Further studies should be undertaken and continuous surveillance periods should provide more information concerning the epidemiology of respiratory virus infections, the pathogenesis and interactions of co-infections and their associations with clinical outcomes.

## Conclusions

We found that respiratory viruses were statistically associated with age and season. Appropriate knowledge and understanding of the clinical symptoms of these respiratory illnesses may contribute to developing better strategies for therapy and prevention.

## Supplementary Information

**Additional file 1:** Geographic regions of respiratory viruses study covered (10 cities, in color area).

**Additional file 2:** Clinical Indicators and Clinical Symptoms Associated with Respiratory Viruses Negative.

## Data Availability

The data and materials supporting the conclusions of the study are available from the corresponding author on reasonable request.

## Abbreviations

ADV: Adenoviruses
ARI: Acute respiratory infections
IFA: Influenza A virus
IFB: Influenza B virus
LRTI: Lower respiratory tract infection
PIV: Parainfluenza virus
RSV: Respiratory syncytial virus
URTI: Upper respiratory tract infection
