# Supplementary figures and images for: Epidemiological and clinical characteristics of respiratory viruses in 4403 pediatric patients from multiple hospitals in Guangdong, China
Source: BMC Pediatr. 2021 Jun 17;21:284. doi: 10.1186/s12887-021-02759-0 (PMC8212487; doi:10.1186/s12887-021-02759-0)

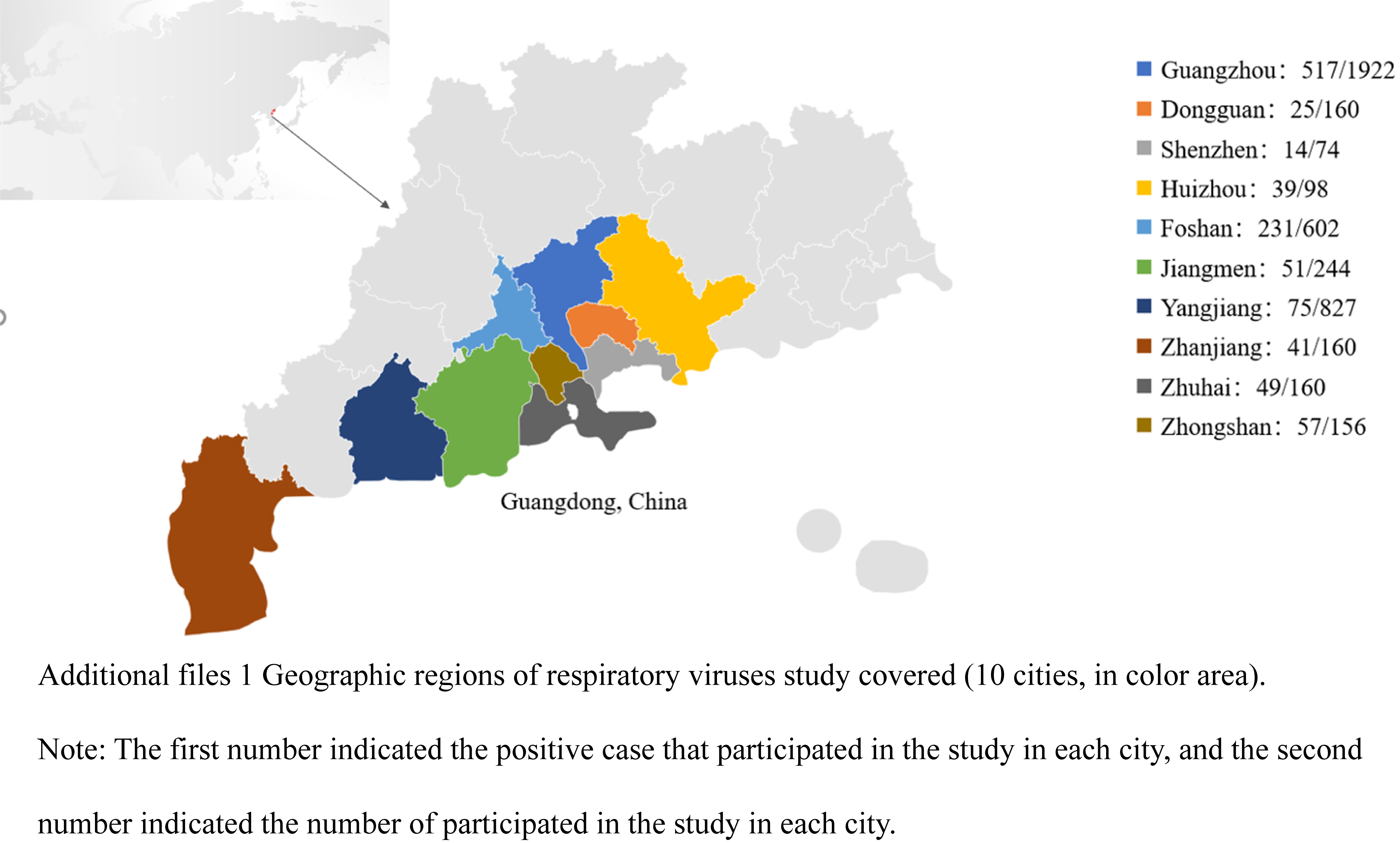

Supplement: Supplementary file 1 — Additional file 1: Geographic regions of respiratory viruses study covered (10 cities, in color area). [file 12887_2021_2759_MOESM1_ESM.tif]
