# Supplementary material for: Epidemiological and clinical characteristics of respiratory viruses in 4403 pediatric patients from multiple hospitals in Guangdong, China
Source: BMC Pediatr. 2021 Jun 17;21:284. doi: 10.1186/s12887-021-02759-0 (PMC8212487; doi:10.1186/s12887-021-02759-0)
Supplement: Supplementary file 2 — Additional file 2: Clinical Indicators and Clinical Symptoms Associated with Respiratory Viruses Negative. [file 12887_2021_2759_MOESM2_ESM.docx]

Additional files 2 Clinical Indicators and Clinical Symptoms Associated with Respiratory Viruses Negative.

| Characteristics | IFA | IFB | ADV | RSV | PIV1 | PIV2 | PIV3 |  |
| --- | --- | --- | --- | --- | --- | --- | --- | --- |
| Clinical indicators | | | | | | | | |
| Respiratory Frequency | 30.03±11.47 | 30.10±11.31 | 30.01±11.33 | 30.00±11.51 | 28.83±10.59* | 30.13±11.40 | 30.02±11.29 |  |
| Days of fever | 4.65±6.27 | 4.67±6.33 | 4.66±6.38 | 4.72±6.50 | 4.71±6.34 | 4.64±6.24 | 4.63±6.31 |  |
| Check temperature | 28.14±16.44 | 28.21±16.41 | 28.08±16.42 | 28.13±16.45 | 27.87±16.60 | 28.33±16.34 | 28.52±16.22 |  |
| Maximum temperature | 31.37±15.77* | 31.61±15.60 | 31.42±15.72 | 31.62±13.90 | 31.59±15.62 | 31.81±15.78 | 31.95±15.34 |  |
| Clinical Symptoms# | | | | | | | | |
| Cyanosis | 16(0.59) | 16(0.57) | 15(0.56) | 11(0.43) * | 15(0.54) | 16(0.57) | 16(0.589) |  |
| fever | 1978(76.10) * | 2057(76.80) | 1981(76.30) * | 1924(77.50) * | 2068(76.80) | 2074(76.80) | 1960(67.50) * |  |
| Shiver | 60(2. 20)* | 67(2.41) | 59(2.21)* | 67(2.64) * | 67(2.41) | 68(2.42) | 68(2.50) |  |
| Nasal congestion | 326(12.10) | 344(12.41) | 327(12.1) | 300(11.73) * | 343(12.40) | 345(12.37) | 328(12.06) * |  |
| Runny nose | 358(13.30) | 374(13.62) | 355(13.20) | 331(12.95) * | 369(13.37) | 373(13.34) | 360(13.24) |  |
| Sore throat | 63(2.34) | 67(2.42) | 65(2.42) | 67(2.62)* | 69(2.50) | 69(2.47) | 69(2.53) |  |
| Cough | 1977(73.49) | 2021(73.36) | 1969(73.42) | 1834(71.98) * | 2015(73.19) | 2043(73.25) * | 1978(72.94) * |  |
| Chest tightness | 8(0.30) | 8(0.03) | 8(0.32) | 8(3.40) | 8(0.31) | 8(0.31) | 8(0.32) |  |
| Shortness of breath | 130(5.24) | 134(5.29) | 123(4.99) * | 113(4.81) * | 135(5.34) | 136(5.30) | 128(5.13) |  |
| Nausea | 13(0.52) | 14(0.55) | 14(0.57) * | 14(0.59) | 13(0.51) | 14(0.54) | 14(0.56) |  |
| Abdominal pain | 47 (1.84) * | 52(1.99) | 53(2.08) | 52(2.14) | 53(2.03) | 53(2.00) | 53(2.07) |  |
| Neurological symptoms | 38 (1.48) * | 43(1.65) | 40(1.58) | 42(1.73) | 43(1.65) | 44(1.67) | 43(1.68) |  |

Note: *: Significant differences between case patients positive and negative for the specific virus (*P*<0.05).

#: case (%)
